# A rare case of proximal closed loop obstruction between gastric band and obstructed ventral hernia

**DOI:** 10.1093/jscr/rjaf326

**Published:** 2025-05-21

**Authors:** Leonid Drober, Ahmad Assalia, Abd Elkarim Darawsha

**Affiliations:** Department of General Surgery, Rambam Medical Center and the Bruce Rappaport, Faculty of Medicine, Technion—Institute of Technology, Rambam Health Care Campus, HaAliya HaShniya St 8, Haifa, 3109601, Israel; Department of General Surgery, Rambam Medical Center and the Bruce Rappaport, Faculty of Medicine, Technion—Institute of Technology, Rambam Health Care Campus, HaAliya HaShniya St 8, Haifa, 3109601, Israel; Department of General Surgery, Rambam Medical Center and the Bruce Rappaport, Faculty of Medicine, Technion—Institute of Technology, Rambam Health Care Campus, HaAliya HaShniya St 8, Haifa, 3109601, Israel

**Keywords:** gastric band, bariatric surgery, closed loop, bowel obstruction

## Abstract

A 68-year-old woman with severe obesity presented with abdominal pain, nausea, and dehydration. She had a history of laparoscopic adjustable gastric banding (LAGB). Examination revealed tachycardia, hypotension, a chronic ventral hernia with mild tenderness, no peritonitis, and slight abdominal distension. A computed tomography (CT) scan showed massive gastric and dilation and obstruction at the level of gastro-esophageal junction (Band), proximal small bowel loop dilation, distal bowel collapse, and a ventral hernia involving high-grade obstruction and ischemic bowel. Exploratory laparotomy revealed a “closed-loop” obstruction between the gastric band and hernia. Her stomach was dilated and obstructed at the gastroesophageal junction, and ischemia affected 70 cm of the intestine in the hernia sac. This case emphasizes that patients with LAGB may be unable to vomit, and therefore a closed-loop obstruction should not be overlooked even without vomiting. Early diagnosis and timely treatment led to a favorable outcome.

## Introduction

Obesity is a significant health issue affecting a large number of people worldwide. Laparoscopic adjustable gastric banding (LAGB) was an effective method for reducing excess weight in obese patients. This procedure is favored by both patients and surgeons due to its advantages, such as a short hospital stay, low mortality rate, effective weight loss, and improvement in comorbid conditions [1, 2]. The overall complication rate, both early and late, is estimated to range from 2.2% to 20% [2, 3]. Small bowel obstruction is a common surgical problem, and its presenting symptoms are well-known, including nausea, vomiting, absolute constipation, abdominal pain, and distention [4].

In patients with a LAGB, vomiting may not occur due to the presence of the band at the esophago-gastric junction. As a result, these patients cannot decompress the obstruction proximally, which, if untreated, may lead to a closed-loop obstruction. The management of small bowel obstruction typically depends on its underlying cause. The management of small bowel obstruction typically involves conservative treatment for adhesions. Closed-loop obstructions or unresolved cases require urgent intervention to prevent complications like bowel ischemia and death [5–7]. In LAGB patients, the risk of closed-loop obstruction makes early diagnosis and intervention crucial. While obstructions between the gastric band and an obstructed ventral hernia are rare, they can be potentially fatal.

We present a case of closed-loop obstruction between the gastric band and an obstructed ventral hernia, where prompt diagnosis and treatment led to a favorable outcome.

## Case report

A 68-year-old woman with severe morbid obesity, hypertension, hyperlipidemia, and an asymptomatic ventral hernia for 10 years presented with a 4-day history of severe abdominal pain, nausea, and dehydration. She had undergone LAGB in 2008, which was replaced in 2015 due to leakage. Her body mass index was 52 kg/m^2^ at the time of the first surgery and 37.5 kg/m^2^ at the time of admission. Physical examination revealed tachycardia, hypotension, large chronic ventral hernia with mild generalized abdominal tenderness, especially over the hernia site, no signs of obstruction or skin changes, and slight abdominal distension, without peritonitis. Laboratory tests showed elevated white blood cells, acute kidney injury, and elevated lactate.

**Figure 1:**
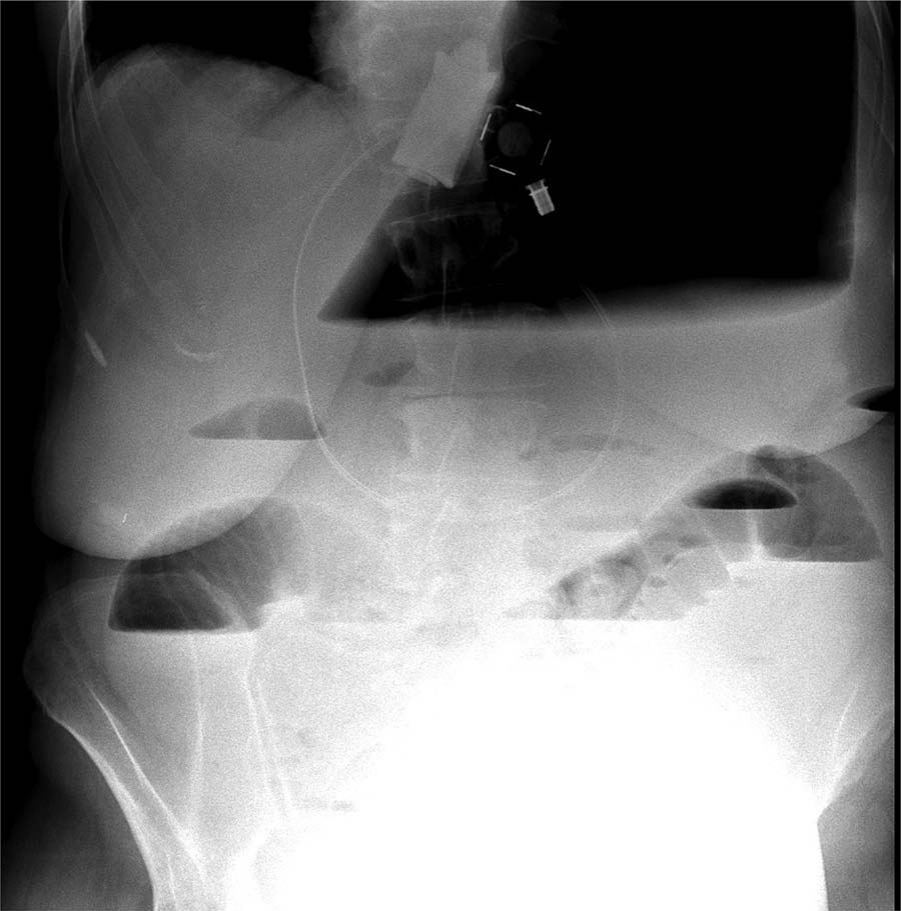
X-ray massive gastric dilatation and small bowel dilatation with air-fluid levels.

An abdominal X-ray revealed that the gastric band was in place, and there was acute massive gastric dilation, which pushed the left diaphragm up. Additionally, there was dilatation of the proximal small intestinal loops with air-fluid levels, indicating bowel obstruction (Fig. 1).

**Figure 2:**
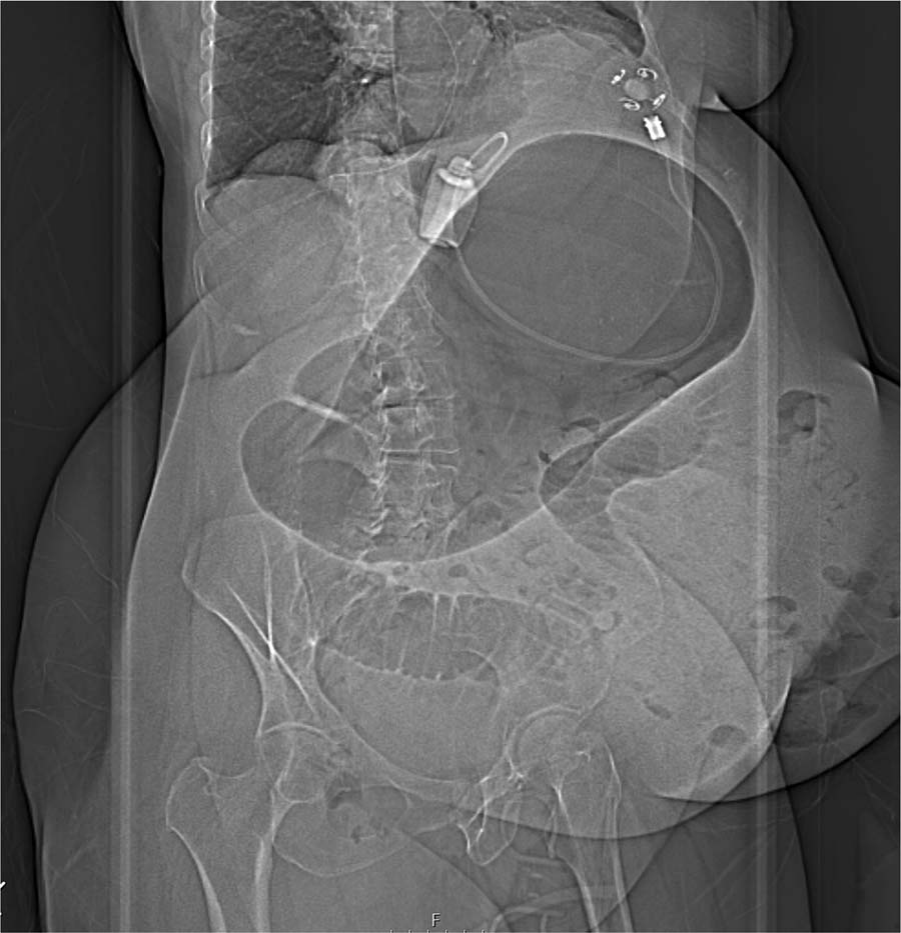
Massive gastric and small bowel dilatation.

**Figure 3:**
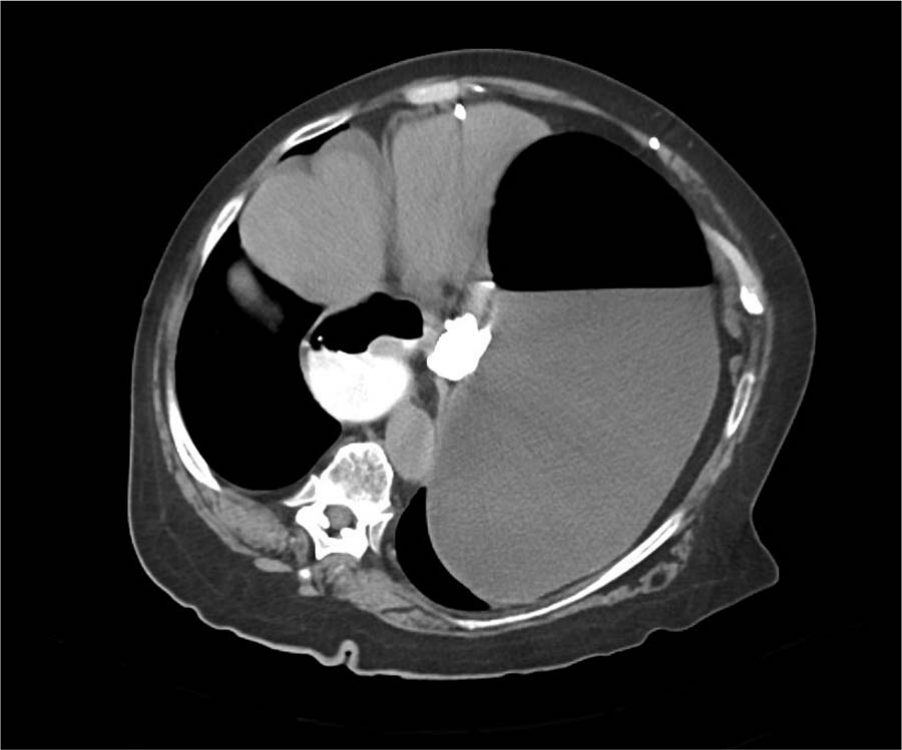
CT axial view—contrast media in distal esophagus did not pass to the stomach.

**Figure 4:**
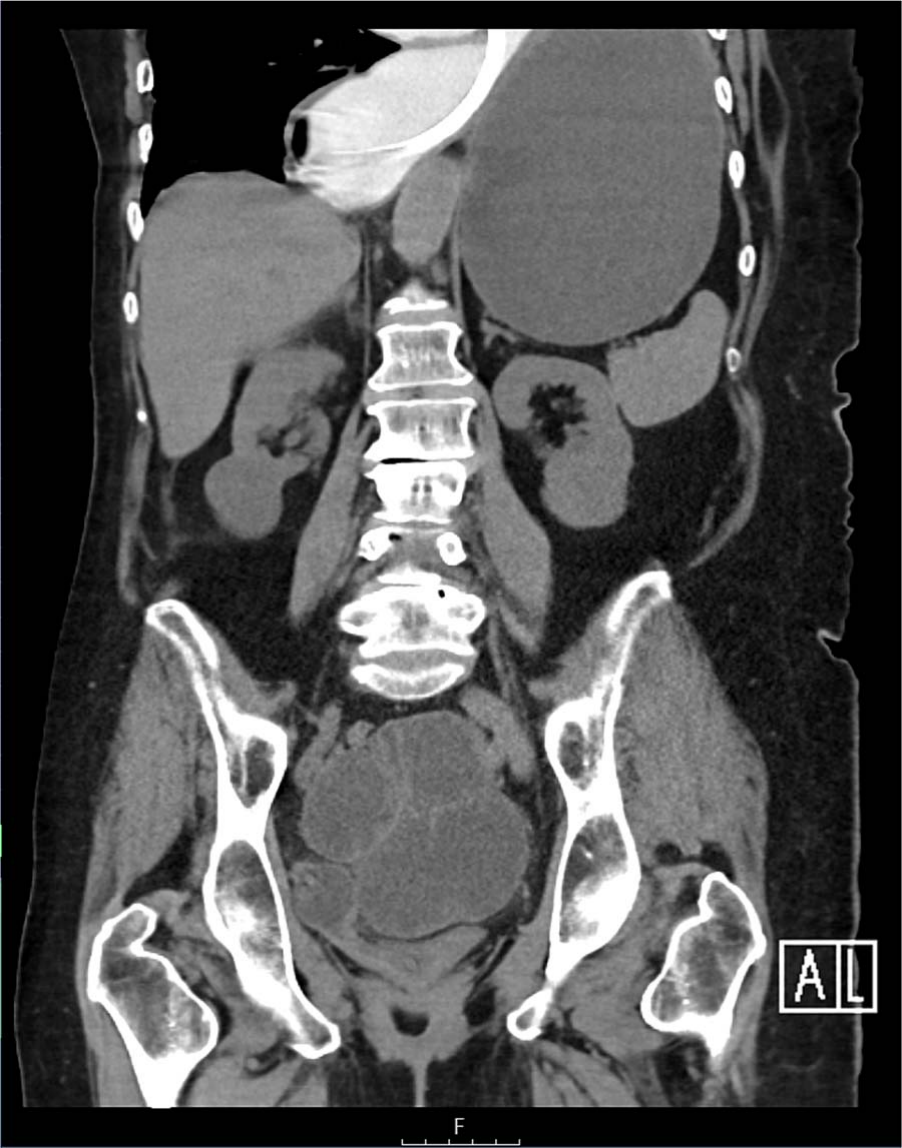
CT coronal view—NGT stuck in distal esophagus.

Immediate action involved removing fluid from the gastric band and inserting a nasogastric tube, which was slightly difficult. Only 200 ml of gastric content was drained, and no air was present (indicating that it was not in the stomach). Given the possibility of anatomical pathology or obstruction at the gastroesophageal junction (GEJ), we avoided excessive force when inserting the tube to prevent iatrogenic esophageal perforation. A computed tomography (CT) scan with liquid-soluble contrast media showed that the contrast did not pass beyond the band. The scan also revealed massive gastric dilatation, as well as dilation of the esophagus proximal to the band (Figs 2–4).

**Figure 5:**
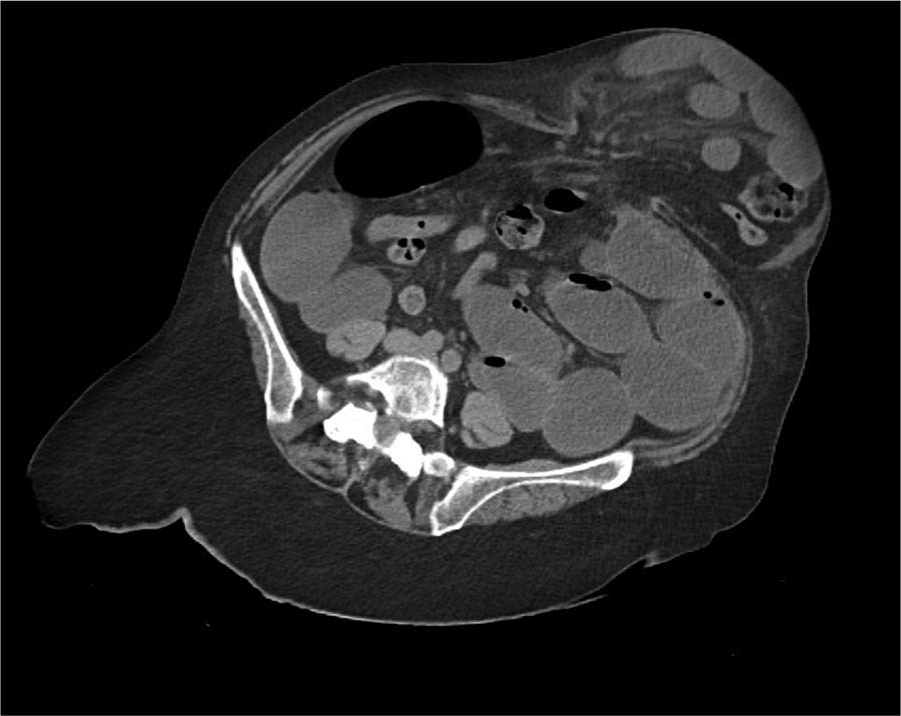
CT axial view—obstructed ventral hernia with proximal small bowel loop dilatation.

The CT also showed a large ventral hernia in the anterior abdominal wall on the left side, with several entrances and exits for intestinal loops within the hernia. The proximal loops appeared dilated up to 5 cm, with fat stranding and slight fluid between the loops, as well as mild prominence of the mesenteric blood vessels. Distal loops were collapsed, raising suspicion of high-grade obstruction with ischemic bowel involvement (Fig. 5).

**Figure 6:**
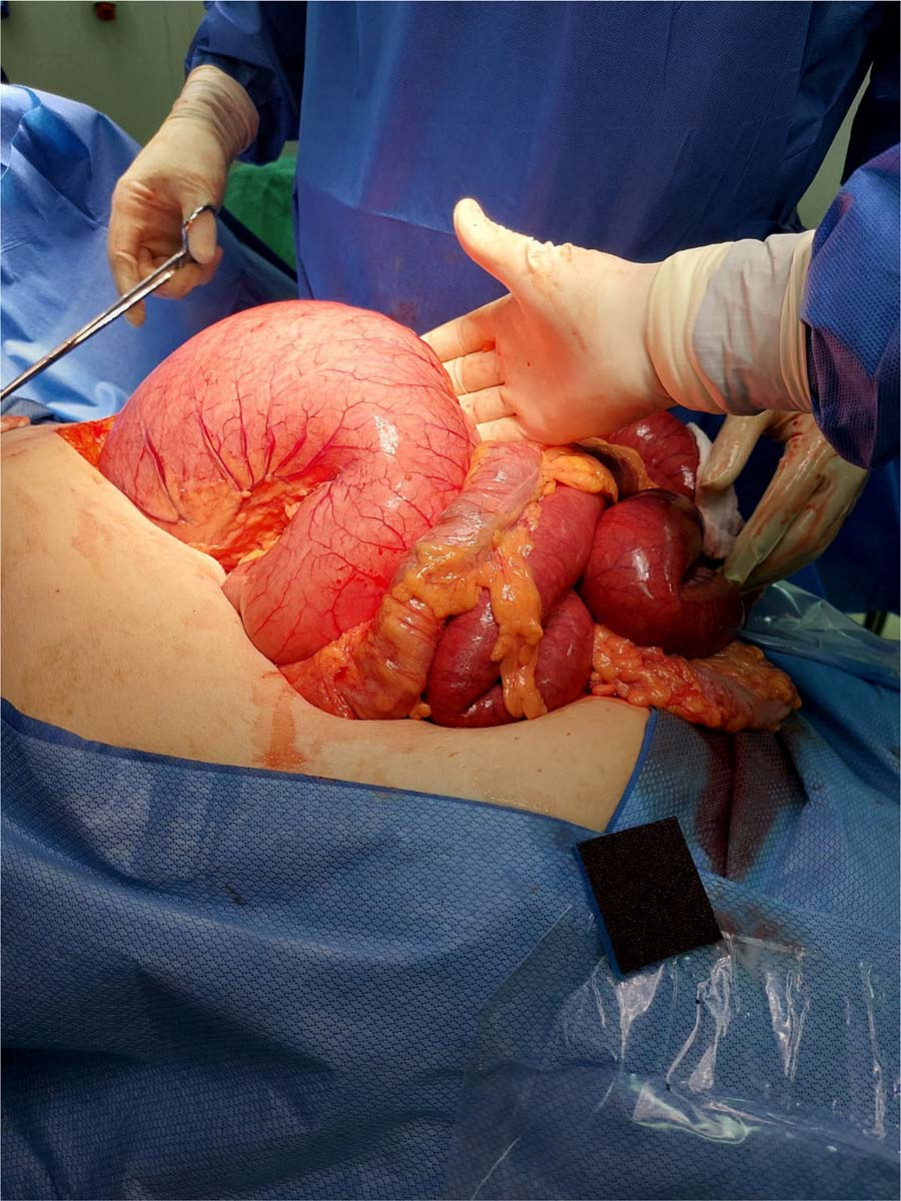
Massive gastric dilatation filled with air and fluid.

Exploratory laparotomy was performed through an upper midline incision. The stomach was found to be distended, filled with air and static fluid (Fig. 6), and obstructed at the GEJ level by the adjustable band (as the nasogastric tube could not pass). Distended jejunal loops proximal to the obstructed ventral hernia, and ischemia of a 70-cm segment of small intestine within the hernia sac. This formed a “closed-loop” obstruction between the band (stomach) and the obstructed ventral hernia (small intestine).

**Figure 7:**
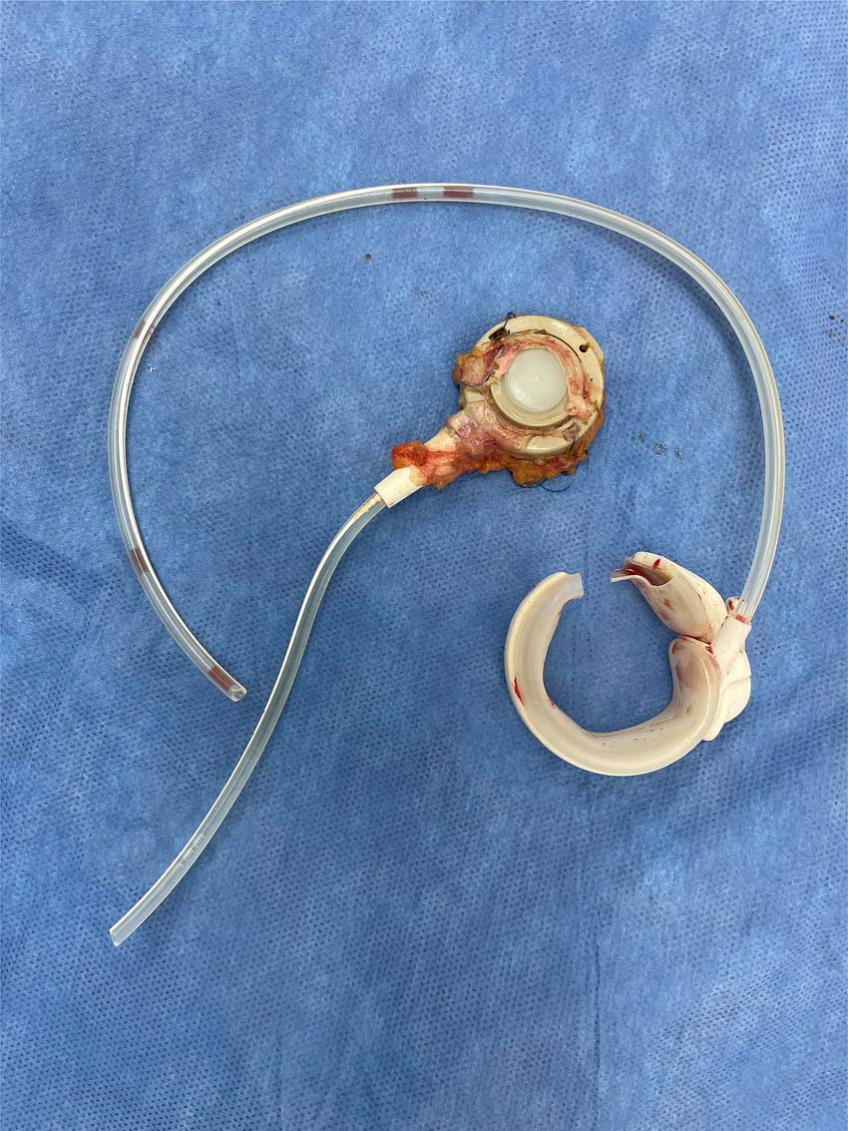
Removed band.

The gastric band was removed (Fig. 7), and a large nasogastric tube was carefully inserted, draining 5 l of fecal content and decompressing the stomach and proximal small intestine. A segmental resection of the ischemic small intestine was performed with a side-to-side primary anastomosis.

After surgery, the patient was hemodynamically stable but experienced respiratory distress and difficulty weaning from the ventilator in the intensive care unit. She also developed ventilator-associated pneumonia, which was treated. There were no anastomotic leaks or wound infections. Slowly patient recovered well, being discharged on postoperative Day 33.

## Discussion

This rare case of gastric distention due to a kink and altered esophago-gastric angle, leading to mechanic esophago-gastric junction outflow obstruction (EGJOO) aggravated by further dilatation of the proximal jejunum, which formed a closed-loop obstruction due to the ventral hernia.

This case emphasizes that patients with LAGB may be unable to vomit, and therefore a closed-loop obstruction should not be overlooked even without vomiting. The symptoms are similar to common LAGB complications, so fluid removal from the band and a barium swallow should be considered for diagnosis. As the stomach distends, it may become impossible to pass a nasogastric tube, likely due to kink/edema and an altered esophago-gastric angle early insertion of a nasogastric tube is crucial to decompress the obstructed stomach and prevent closed-loop obstruction. If the tube cannot be passed, early surgery is necessary [7].

Known complications of LAGB include esophageal dilation, intragastric band erosion, gastric perforation, band slippage, abscess formation, tube disconnection, port-site infection, and small bowel obstruction [8–12]. Respiratory complications, such as aspiration pneumonia, pulmonary abscess, and empyema, are also documented [13, 14]. Small bowel obstruction is considered a complication of LAGB only when attributed to adhesions in patients without prior surgical history. This is the first case of such a rare closed-loop pathology documented.

## Conclusion

Obstructions between the gastric band and an obstructed ventral hernia are rare but potentially fatal. This case of massive gastric dilatation due to a kink and altered esophago-gastric angle, leading to EGJOO and aggravated by proximal jejunal dilation from the ventral hernia, resulted in a closed-loop obstruction. Early removal of fluid from the band and insertion of a nasogastric tube are critical. If a nasogastric tube cannot be passed, early operative intervention should be considered. In our patient, early diagnosis and prompt treatment resulted in a favorable outcome.

## References

[1] Richardson J, Smith B. Laparoscopic gastric banding. Minerva Chir 2012;67:141–52. 22487916

[2] Eid I, Birch DW, Sharma AM, et al. Complications associated with adjustable gastric banding for morbid obesity: a surgeon’s guide. Can J Surg 2011;54:61–6. 10.1503/cjs.015709 21251434 PMC3038361

[3] Alhamdani A, Wilson M, Jones T, et al. Laparoscopic adjustable gastric banding: a 10-year single-centre experience of 575 cases with weight loss following surgery. Obes Surg 2012;22:1029–38. 10.1007/s11695-012-0645-9 22488681

[4] Tjandra JJ, Clunie GJA, Thomas RJS. Textbook of Surgery. Blackwell Publishing, 2001.

[5] Fevang BT, Fevang J, Stangeland L, et al. Complications and death after surgical treatment of small bowel obstruction: a 35-year institutional experience. Ann Surg 2000;231:529–37. 10.1097/00000658-200004000-00012 10749614 PMC1421029

[6] Attard JAP, MacLean AR. Adhesive small bowel obstruction: epidemiology, biology and prevention. Can J Surg 2007;50:291. 17897517 PMC2386166

[7] Campbell NA, Brown WA, Smith AI, et al. Small bowel obstruction creates a closed loop in patients with a laparoscopic adjustable gastric band. Obes Surg 2008;18:1346–9. 10.1007/s11695-008-9622-8 18654823

[8] Colquitt JL, Pickett K, Loveman E, et al. Surgery for weight loss in adults. In: Colquitt JL (ed.), Cochrane Database of Systematic Reviews. Chichester: Wiley, 2014. 10.1002/14651858.CD003641.pub4 PMC9028049 25105982

[9] Sonavane SK, Menias CO, Kantawala KP, et al. Laparoscopic adjustable gastric banding: what radiologists need to know. Radiographics 2012;32:1161–78. 10.1148/rg.324115177 22787000

[10] Blachar A, Blank A, Gavert N, et al. Laparoscopic adjustable gastric banding surgery for morbid obesity: imaging of normal anatomic features and postoperative gastrointestinal complications. AJR Am J Roentgenol 2007;188:472–9. 10.2214/AJR.05.0293 17242257

[11] Levine MS, Carucci LR. Imaging of bariatric surgery: normal anatomy and postoperative complications. Radiology 2014;270:327–41. 10.1148/radiol.13122520 24471382

[12] Shen X, Zhang X, Bi J, et al. Long-term complications requiring reoperations after laparoscopic adjustable gastric banding: a systematic review. Surg Obes Relat Dis 2015;11:956–64. 10.1016/j.soard.2014.11.011 25638595

[13] Savir S, Kalchiem-Dekel O, Naggan L, et al. Respiratory deterioration following laparoscopic adjustable gastric banding: a three year follow-up of over 3,000 subjects. Respir Med 2016;115:66–71. 10.1016/j.rmed.2016.04.014 27215506

[14] Avriel A, Warner E, Avinoach E, et al. Major respiratory adverse events after laparascopic gastric banding surgery for morbid obesity. Respir Med 2012;106:1192–8. 10.1016/j.rmed.2012.05.002 22673900

